# Exploring trainee experiences in a structured virtual reality laparoscopic training programme for general surgeons: a longitudinal case study

**DOI:** 10.1186/s41077-025-00359-x

**Published:** 2025-10-28

**Authors:** Aditi Siddharth, Sotiris Mastoridis, Michael Silva, Debbie Aitken, Helen Higham

**Affiliations:** 1 Oxford Simulation, Training and Research Centre (OxSTaR), University of Oxford, Oxford, UK; 2 General Surgery, Oxford University Hospitals, Oxford, UK; 3 Department of Education, University of Oxford, Oxford, UK

**Keywords:** Simulation based education, Surgical training, Virtual reality simulation, Laparoscopic training, Technical skills training, Qualitative research

## Abstract

**Background:**

The acquisition and maintenance of technical skills in surgical specialties has become increasingly challenging for postgraduate trainees, exacerbated by factors such as the shift from traditional apprenticeship models, reduced operative time, and the impact of the COVID-19 pandemic. Virtual reality (VR) simulators offer a promising adjunct to traditional surgical training, though their integration into routine practice remain underexplored.

**Objective:**

This qualitative study investigates the experiences and motivations of general surgical trainees who engaged with a VR laparoscopic simulator as part of a structured training program.

**Methods:**

A case study methodology was chosen to explore the experiences of 22 general surgery trainees using a VR laparoscopic simulator over a period of 3 months. Each of the trainees were adviced to practise a minimum of five repetitions across 25 laparoscopic simulator exercises. The study was designed using Kopta’s theory of technical skill learning, focusing on the cognitive phase, where trainees repetitively practised individual steps with feedback. Data collection involved qualitative questionnaires, semi-structured interviews (of seven of the trainees, 8 months later), and quantitative data from the simulator. The qualitative data was analysed using thematic analysis, and descriptive statistical tests were applied to the quantitative data for triangulation.

**Results:**

The study identified key factors influencing trainee engagement, including ease of access, the importance of periodic rather than frequent simulation sessions, Annual Review of Competency Progression (ARCP) overview and the value of setting specific performance goals. The findings suggest that simulation can effectively complement traditional surgical training when incorporated into routine practice, with potential for broader application if barriers such as time constraints and access issues are addressed.

**Conclusion:**

This study contributes to the literature on surgical education by highlighting the need for targeted strategies to enhance the use of simulation as an adjunct alongside more traditional training.

**Supplementary Information:**

The online version contains supplementary material available at 10.1186/s41077-025-00359-x.

## Background

Postgraduate surgical trainees increasingly report challenges in acquiring and maintaining technical skills in surgical specialities [1, 2]. Factors contributing to this include a shift from the apprenticeship model, reduced operative exposure, working time restrictions, rising patient complexity, advancements in surgical techniques, and the impact of the COVID-19 pandemic [3, 4]. The pandemic, in particular, led to demotivation and reduced engagement in the collaborative learning process [5–7]. Studies indicate that trainees often feel underprepared and less independent in their surgical skills upon completing training [8–11], prompting many to pursue additional training before assuming consultant roles [12, 13].

The use of simulation training as an adjunct to workplace learning of technical surgical skills is evidence based [14–16]. Irrespective of the device tested, whether that be laparoscopic box trainers, simple low fidelity simulation models or extended reality environments including virtual reality (VR), evidence shows they are effective in reducing the learning curve, improving efficiency of operating, reducing surgical complications, and improving patient safety [16–20]. The majority of the studies demonstrating efficacy of surgical simulation technical skill training were conducted in study conditions for a short time period, often with a “boot camp” like structure where pre and post training scores were compared to show improvement of skills [21–24]. All efforts to improve surgical training including the “Improving Surgical Training (IST)” initiative in the UK recommended the regular use of simulation for developing both technical skills and non-technical skills [25, 26]. Despite national level interest and the recognition of its value by policy makers, there have been significant challenges with implementing regular simulation training at a curricular level as well into practice [27–29]. The challenges in the UK and globally, include the paucity of time in a busy trainee’s work schedule, inadequate numbers of faculty trained to deliver simulation-based education, difficulty with planning and motivating trainees to attend regular simulation sessions [30–32]. Concerns about the cost involved with setting up a simulation centre may also inhibit its use [30].

The implementation of simulation as a regular adjunct to clinical training is an under researched area. Understanding the trainees’ experience and the factors that motivate the regular use of the available facilities should help educators and educational researchers innovate to enhance the use of available simulation facilities cost effectively, efficiently and without an undue increase in resource burden [33, 34].

**Fig. 1:**
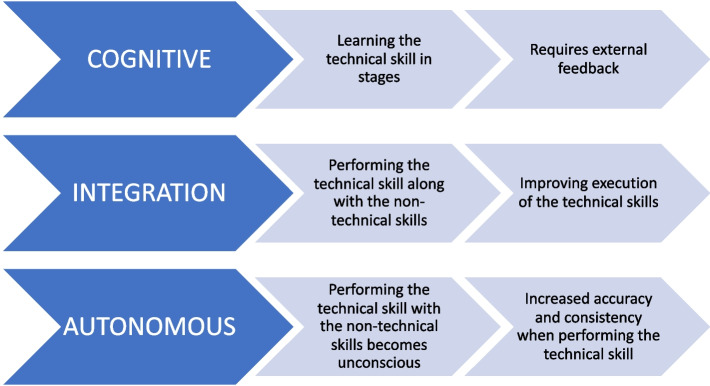
Kopta’s three stages of technical skill learning [35]: cognitive, integration and autonomous. This theory helps explain the phases of learning any technical skill and has previously been applied to learning to perform a surgical procedure in the operating theatre

To address this gap in literature, we investigated a simulation training programme with a VR simulator that trainees had 24/7 access to, with specific targets to motivate regular use alongside their routine training. We used Kopta’s educational theory for learning technical skills (Fig. 1) as the theoretical basis for designing this study [35]. Our research question was: *What is the trainee experience of learning technical skills in the cognitive phase using simulation*? Our aim was to understand the trainees’ existing experiences with using simulation to learn technical skills and the impact of a structured programme that they could access alongside their routine training.

## Methods

### Context

In the UK, surgical training progresses from a 2-year core training (CT1-CT2) to a 6-year higher speciality programme. Trainees rotate through hospitals within a Postgraduate Deanery (PGD), which oversees training and reviews progress through the Annual Review of Competency Progression (ARCP). Technical skills are primarily learned in the workplace under supervision, supplemented by PGD-organised training, including simulation. This study, conducted in the Thames Valley PGD, involved ST3 + trainees. It explored their views on different simulation methods and their comparison to the structured programme and VR simulation used in this study. The study was registered as a quality improvement project with Oxford University Hospitals and received ethics exemption from the Clinical Trials and Research Governance (CTRG) unit, University of Oxford.

### Methodology

This qualitative study is underpinned by the interpretivism paradigm acknowledging that reality is subjective, constructed by individuals based on their interactions with the people and objects and may be perceived differently based on their context, history, culture, and experiences. This philosophical positioning aligned well with the chosen methodology of case study [36]. The instrumental case in this study was the structured VR simulation programme that trainees accessed alongside their routine training. The emphasis was on using a pragmatic approach; the general surgical education team had acquired the VR device for trainees and the structured programme was designed to fit around regular training. We chose a qualitative approach as it allowed for the capturing of deeper insights of the trainees’ experiences using qualitative questionnaires and semi structured interviews. The theoretical framework used to design the study was Kopta’s theory of technical skill learning [35]. Kopta describes three stages to learning a technical skill: cognitive, integrative, and autonomous. In the cognitive phase, the learners practice repeatedly the individual steps of a technical skill receiving feedback to correct technique before they can combine them in sequence and perform the steps all together. In the integrative and autonomous phases, the technical skill is performed in the context where it used, (in this case the operating theatre), along with the non-technical skills required to deliver increasingly smooth performance of the technical skill so that the trainee has to think less and less about the steps involved and the performance of the technical skill becomes unconscious. In this study, the simulation was designed for the surgeon to practise the technical skill (laparoscopic operating) in the cognitive phase, with the simulator providing regular feedback using scores to track progress.

### Participants

All the ST3 to ST8 trainees in the Thames Valley deanery were asked to attend the mandatory regional training day on June 27th, 2022 (convenience sampling). Anyone who could not attend the regional training due to on call commitments or annual leave was asked to inform the training programme director via email and was excluded from the study though they had access to the simulator for the study period. We discussed the length of the study, the data collection methods used and the time commitment that would be required of the trainees, provided a written information sheet for them to consider and took written consent for participating in the study. The trainees who participated in the study were contacted 8 months after the end of the study for a follow-up face to face semi structured interview (purposive sampling) until thematic sufficiency was achieved.

### The simulator used for the study

**Fig. 2:**
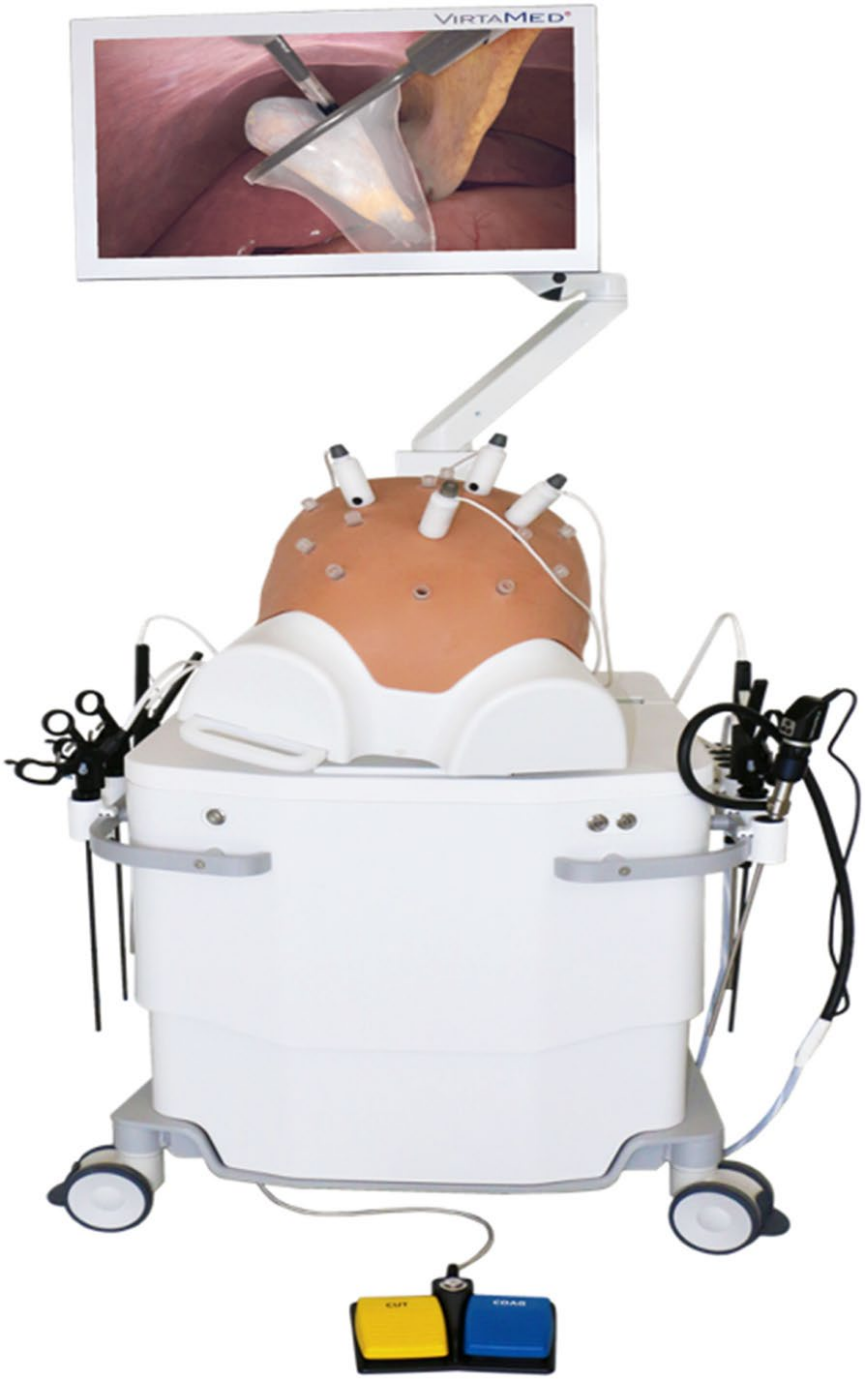
The VirtaMed™ Virtual Reality (VR) simulator. Image provided by VirtaMed™. The simulator has a model similar to the inflated abdomen seen during laparoscopic surgery, port sites to insert physical instruments, diathermy pedals, and a screen. The simulator provides some haptic feedback when used, giving the user a realistic sensation of touching structures as you would experience when performing laparoscopic surgery in real life

**Fig. 3:**
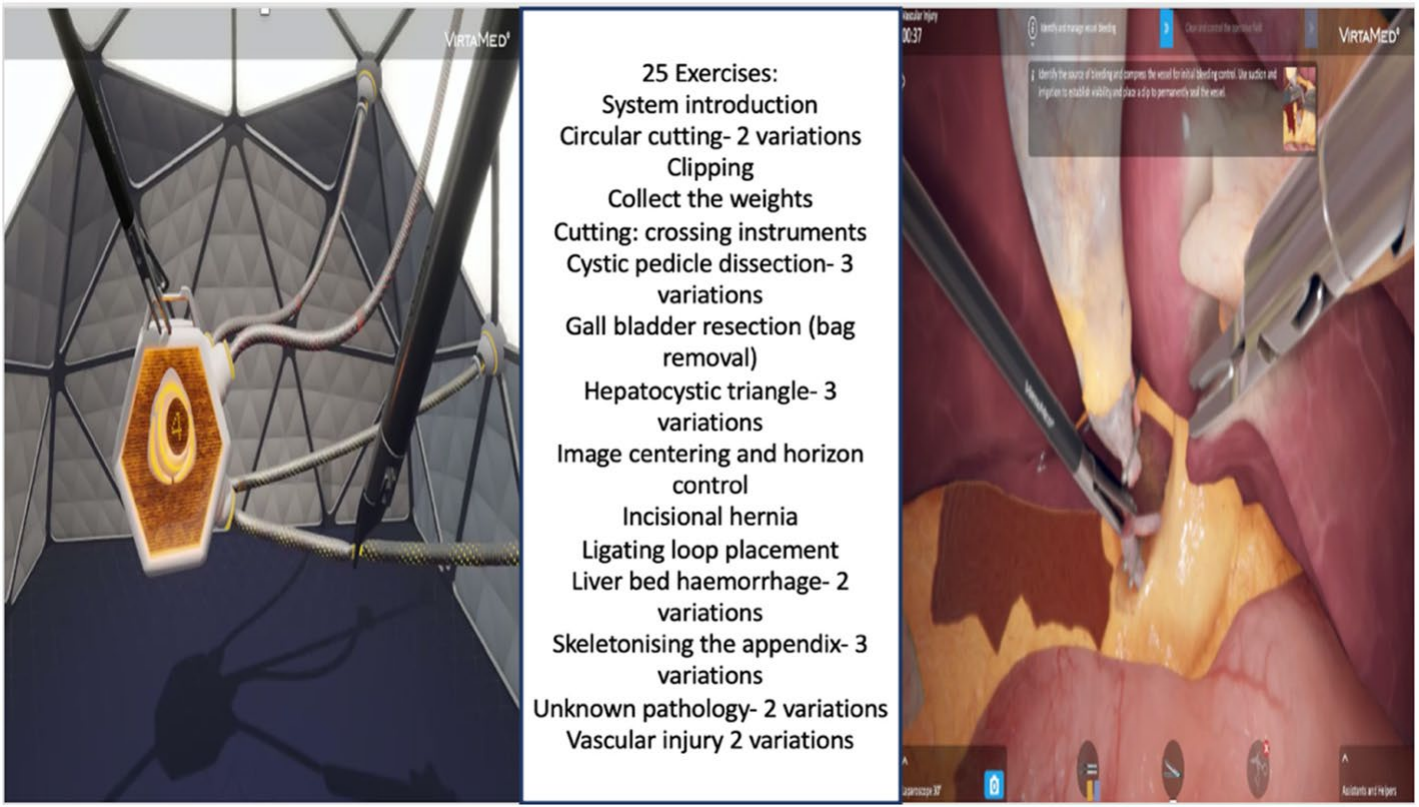
The two interfaces for exercises on the VirtaMed™ VR simulator- gamified and anatomically accurate. Twenty-five exercises were available to the participants of the study- with eight gamified exercises and 17 anatomically accurate exercises. The anatomically accurate exercises showed the anatomy of the liver, gall bladder hepatocytic triangle, etc. depending on the exercise the user chose to perform

The laparoscopic simulator was provided by VirtaMed ™ for the duration of the study (Fig. 2). The simulator had three parts: a hard plastic casing resembling an abdomen inflated with carbon dioxide (as usual in a laparoscopic operation) into which instruments could be introduced; two laparoscopic instruments; and a monitor which displayed the virtual abdominal contents that allowed the operator to perform the exercises. The instruments could move realistically and provided haptic feedback. The choice of instrument could be made by choosing from a list on the screen (scissors, grasper, diathermy, etc.) rather than needing to change it physically. The exercises available to the trainees had two interfaces- gamified and anatomically accurate (Fig. 3). The simulator at the completion of an exercise, provided the trainee with an overall score, a breakdown of the score as well as feedback on how to improve their performance. The simulator was placed in the Churchill Hospital (which is part of the Oxford University Hospitals NHS Foundation Trust in Oxford, UK) in a room at that could be accessed 24/7 by the trainees.

### The simulation programme

Trainees attended their mandatory regional study day, during which time they were set up with a log in for the simulator, performed a baseline assessment on the simulator and were shown how to access the simulator any time they wanted to practise on it. The trainees were advised to perform five repetitions on at least of the 25 exercises they had access to over a duration of 3 months (Fig. 3). A week after their baseline assessments, the trainees were emailed a baseline qualitative questionnaire (see Supplementary material 1) that enquired about their previous simulation experience, the impact of the pandemic on their training and self-reported confidence for managing the various aspects of their role that involved technical skills and setting personal objectives for the training they were about to experience. A midpoint review of engagement with the simulator was conducted and the trainees were reminded about increasing their engagement. At the end of the 3 months, the trainees completed a follow-up assessment on the simulator. They were emailed a qualitative questionnaire as a follow-up (see Supplementary material 1) which enquired about the self-reported impact of the simulator use on individual technical skills, their experience using the VR simulator (both successes and challenges), their views on where this structured programme with a self-access simulator would best fit with their training as well as whether their personal objectives had been achieved. During the 3-month training period, the trainees continued with their regular training and work commitments. They were encouraged to use their study sessions and study leave if required to use the simulator. They continued with their routine regional training days both during the 3 months as well as after, having access to other forms of simulation, e.g. the laparoscopic box trainer (located in their local hospitals), robotic simulator (located at the Churchill hospital) and augmented reality (AR) simulator (which they practised on during a regional study day). Their engagement with the VR simulator was reviewed at the end of 3 months during their ARCP meeting. The trainees who used the VR simulator were invited to participate in follow-up semi structured interviews (see Supplementary material 1 for the interview guide), 8 months after the completion of the 3-month engagement with the VR simulator to understand the long-term role simulation might play in their training.

### Data collection

We developed two qualitative questionnaires and an interview guide for the follow-up semi-structured interview using best practice advice (provided by Boynton et al.) to answer our research question and understand the trainee’s simulation experience, their wishes on how simulation may be best used to enhance their technical skill training, approaches that might motivate them to engage further with simulation etc. [37]. All of the interviews were conducted face to face, recorded and transcribed before analysis. The lead researcher who conducted the semi-structured interviews also maintained a research diary to record observations such as body language and facial expressions etc. The questionnaires were sent and collected electronically, giving the trainees time to consider their answers for each of the qualitative questions. Quantitative data from the simulator was also available for analysis and was used to triangulate the qualitative findings.

### Data analysis

The interviews were digitally recorded, transcribed and the participant data were anonymised. Similarly, the data from the qualitative questionnaires were anonymised. All the qualitative data were analysed using NVivo software to facilitate data management and coding. We conducted a thematic analysis to develop themes and subthemes. The coding was conducted iteratively, using an inductive process, and focussing on answering the research question. Context was discussed among the research team to ensure the themes developed were representative of the general surgical experience as well the theory supporting simulation for technical skills training. The themes developed helped provide insight into best practices to implement simulation into the busy lives of a surgical trainee. We used the paper by Naeem et al. as a guide when performing the thematic analysis, including the development of abstract concepts using the qualitative data for the development of themes and subthemes [38]. The quantitative data collected from the simulator was analysed using a range of statistical tests. Descriptive statistics was used to summarise the data. Further statistical analysis with a t-test, non-parametric tests for correlation analysis were used to assess the relationship between variables helping to contextualise and triangulate the qualitative findings.

### Reflexivity and rigour

The members of the research team had different clinical and research backgrounds (general surgery/obstetrics and gynaecology/anaesthesia and qualitative/quantitative research experience). All were senior in their respective fields and had experience with organising and delivering simulator training. The team regularly discussed their positionality and the biases of their experiences and the influence of the interpreting of the results, relying on the wider team and an iterative process to maintain rigour. The quantitative provided an additional source of triangulation to enhance the data analysis and interpretation.

## Results

Twenty-two general surgical trainees participated in the study. Seven of the trainees from this cohort were interviewed 8 months later (denoted as trainee 1 to trainee 7 in Table 1). The trainees had worked in five different hospitals in the Thames Valley region, representing all the hospitals in the region. The interviews ranged between 20 and 30 min each and all transcripts were utilised in the analysis, along with the qualitative questionnaires, the quantitative data from the simulator and research diary observations. The interviews were continued until thematic saturation was achieved. Participant identities were anonymised, and each participant was assigned pseudonyms so that their questionnaire responses, interview data and quantitative simulator data could be compared for context. Once the qualitative data was coded and themes emerged, the quantitative data was analysed keeping the themes as a point of reference to use as a means of triangulation, to compare and contrast to the qualitative data. There were no trainees in ST8 who took part in this study. There was near equitable distribution amongst the other training years from ST3 to ST7 (Table 1). The distance trainees travelled to access the simulator ranged from 0 miles (trainees who worked on site) to 40 miles, which was the furthest hospital with an average of 18 miles travelled (Table 1). Only one trainee reported not being on track with their training.

**Table 1:** Table presenting the specialist training (ST) year of each participant, the frequency of simulator use over a 3-month period (number of times exercises were performed; higher values indicate greater engagement), and the distance in miles from the trainee’s workplace to the simulator. Baseline and final performance scores are shown for each trainee, where lower scores reflect poorer performance, and higher scores indicate better performance. The improvement score represents the difference between the final and baseline scores (final–baseline)

Trainee no	ST year	Simulator use in the past 3 months (no. of sessions)	Distance to simulator (miles)	Baseline score	Final score	Score improvement (final–baseline score)
1	5	80	0	631	7071	6440
2	5	28	0	373	444	71
3	5	167	0	937	16,832	15895
4	4	69	37	379	5469	5090
5	3	7	37	359	359	0
6	4	23	40	377	1979	1602
7	6	11	22	382	973	591
8	3	13	22	368	1621	1253
9	4	81	0	287	7145	6858
10	5	25	22	340	1288	948
11	6	35	28	362	1687	1325
12	7	17	28	395	1896	1501
13	4	45	40	386	2807	2421
14	6	3	28	367	367	0
15	7	7	22	317	453	136
16	7	12	0	391	606	215
17	5	13	0	391	1098	707
18	6	37	28	383	3017	2634
19	3	68	0	387	7116	6729
20	5	31	0	136	1542	1406
21	3	84	0	385	9858	9473
22	4	34	40	376	3417	3041
Mean		40.45	17.9	395.86	3502.04	3106.18 (*p* = 0.00064)

### The thematic analysis

**Fig. 4:**
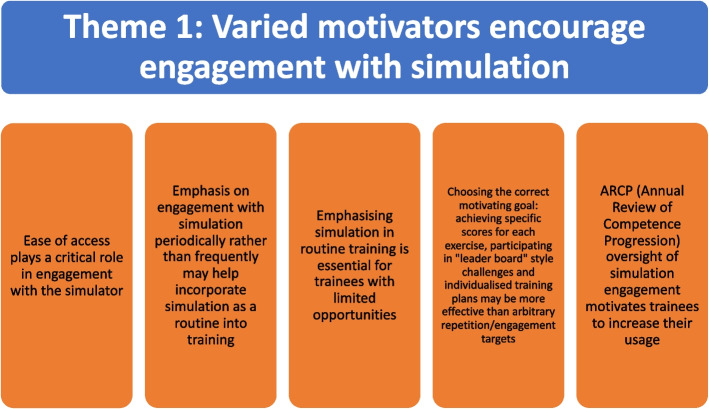
Results of the thematic analysis. Theme 1 with subthemes

**Fig. 5:**
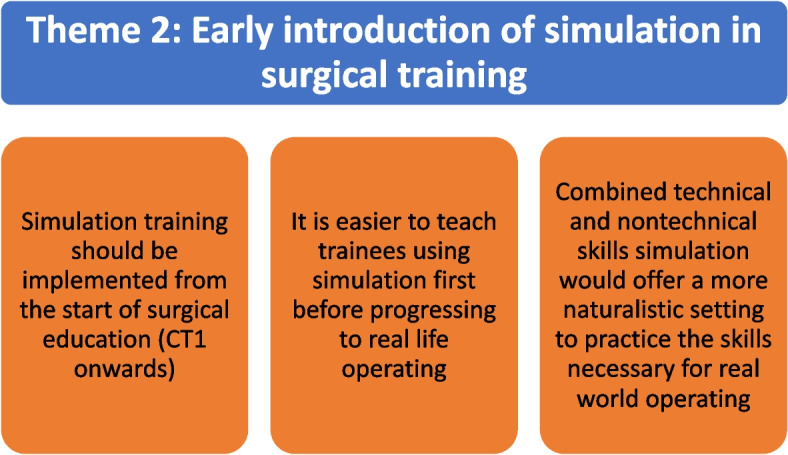
Results of the thematic analysis. Theme 2 with subthemes. CT1: Core surgical training year 1

There were two themes and eight subthemes that emerged from the qualitative questionnaires and interview transcripts (Figs. 4 and 5). The quantitative data was analysed after the thematic analysis and has been presented alongside to triangulate the qualitative data and provide context for interpretation.

### Theme 1: Varied motivators encourage engagement with simulation (Table 2)

**Table 2:** Theme 1: Varied motivators encourage engagement with simulation and the 5 subthemes

Subtheme	Example excerpts from questionnaires (Tr-trainee/participant)	Example excerpts from follow-up interviews (Tr-trainee/participant)
1. Ease of access plays a critical role in engagement with the simulator	Tr 8: “Ease of access – getting to Churchill” Tr 12: “Access due to distance would not have been a problem if I was working in Oxford” Tr 14: “The site being only in Oxford” Tr 18: “Access, it took me two hours round trip to get to and from the hospital” Tr 22: “Getting to the Churchill when working a long distance away, and the costs and time this entailed”	Tr 1: “But I do think there are things I can gain from appropriate simulators. If I feel they are readily available, sure.” Tr 3: “It’s because I was local here. I just used it.” Tr 5: “Improving access. People would be encouraged”
2. Emphasis on engagement with simulation periodically rather than frequently may help incorporate simulation as a routine into training	No corresponding data from the questionnaires for this sub- theme	Tr 3: “I think initially if you want to master it, the more you use it the better. Then maybe you can reduce the number of sessions once you are happy, to maintain skills” Tr 5: “ I understand that’s something that should be integrated into our teaching. I think probably you’ll see benefits proportional to the amount you use” Tr 7: “The complication rate didn’t necessarily change whether you did the case twice a week or twice a month. I think ideally I would want regular sessions to use the simulator within the timetable. As part of training. Like u would have clinical endoscopy, my next morning is my dedicated simulation session.”
3. Emphasising simulation in routine training is essential for trainees with limited opportunities	No corresponding data from the questionnaires for this sub- theme	Tr 1: “I think it’s important for people coming onto jobs like upper gastrointestinal or any jobs that need intracorporeal suturing, to develop that (by practising on a simulator)” Tr 2: “As a middle to senior trainee, if I am doing a job where I’m getting a lot of laparoscopic exposure maybe I don’t need to take an evening to come spend with the simulator. You actually miss it when you’re doing a non-laparoscopic job and it’s been a long time since I’ve done this, I should practice. 100% will benefit people who are not in laparoscopic jobs” Tr 3: “I was just doing it when I had time, going there, suturing, suturing, suturing (on a simulator). Next year, I went to upper gastrointestinal firm, and when they had suturing on their anti-reflux procedures, I noticed you know I was doing it without any problems. The consultants could see I can do it, fine do it” Tr 7: “If your firm is not doing anything then I think yes”
4. Choosing the correct motivating goal: achieving specific scores for each exercise, participating in "leader board" style challenges and individualised training plans may be more effective than arbitrary repetition/engagement targets	Tr 2: “I did not like repetitive activities and rigid in the steps to complete the exercise in the virtual reality simulator” Tr 3: “It (the simulator) also gives feedback so they (the trainee) can act on feedback and try to improve by repeating exercise” Tr 7: “I enjoyed the gamified exercises. It was really fun” Tr 15: “Some of the tasks were interesting. It was useful to keep a score on performance”	Tr 1: “It has to be structured. People won’t use stuff if they aren’t incentivised to use it. So, I think if you say you need to clock up x number of hours and you also need to get scores over a certain number then I think they’ll just be able to do more work on it. And it’ll be to their benefit, I’m sure.” Tr 3: “It is nice to have some structure especially initially when you start, and you are a novice. But I think once again when u have done all the structured training, so then it’s nice to have some variety and some self-direction.” Tr 7 “I think, especially as surgeons, we tend to want to do well with the marks. With the actual tasks I would score really high. I think the simulation should be more tailored to what level you are at and what you have done in real life and then tailor it to the person and what you’ve done in real life. On the robotic simulator and I quite like that. There are lots of exercises you can do and the target there is u need to achieve 90% and above for each of the exercises and until you have done that you cannot go on to the next step or you should really do that before u do any live surgery. It considers that everybody learns at different rates and some people can get 90% just after 5 goes and others might need 30, so in a way it doesn’t matter how many times you do it as long as u achieve that 90% and over.”
5. ARCP (Annual Review of Competence Progression) oversight of simulation engagement motivates trainees to increase their usage	Tr 7: “ARCPs should have an element of simulation for assessment. Maybe review the video of trainee doing the simulation or their engagement with the simulator or the scores they have got over time on the simulator.”	Tr2: “I appreciate the reason for a structured programme and the reality is people are less likely to use it if it’s always just there” Tr 4: “On a simulated model, I appreciate and accept that other people might find it incredibly useful to show it to a consultant or a senior for feedback would be really good. I’d like to see data for myself and for a collection of trainees over the entirety of training, progression through the years.” Tr 7: “From the ARCP, you should have goals for this year from the simulation side.” what you want to improve on. Let’s see if by the end of the year you are able to achieve that. Then it’s your personalised target and goals for simulation”

Different participants were motivated by a variety factors to engage with simulation in a consistent fashion, and this can change over the course of their training programmes. Some motivators, including the use of a target score to be achieved for each exercise before moving on, were a common idea shared by the majority of participants.

#### Ease of access plays a critical role in engagement with the simulator

**Fig. 6:**
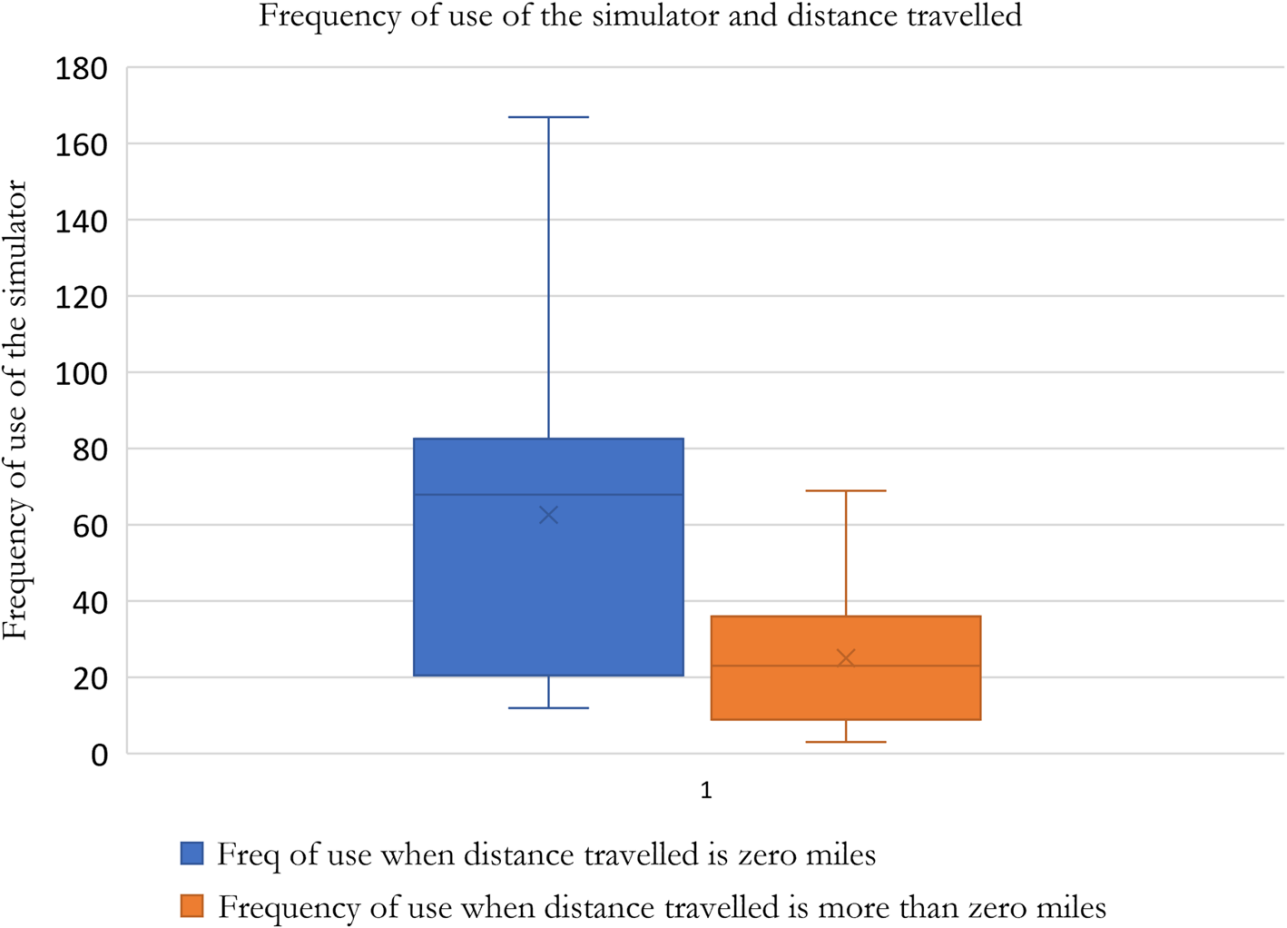
Box and whisker plot illustrating the frequency of simulator use based on the distance travelled. The blue box represents the frequency of use when the distance travelled is zero miles, showing a higher median frequency and greater variability compared to the orange box, which represents the frequency of use when some distance (more than zero miles) is travelled. The median frequency of use is lower in the latter group, with less variability and a smaller interquartile range (centre bar: median, hinges: 1st and 3rd quartile, whiskers: 1.5 × interquartile range, points: outliers). A statistically significant difference was found between the two groups (*p* = 0.045). Frequency of use of the simulator indicates the number of times exercises were performed on the simulator by a trainee. The higher the value of frequency of use indicated better engagement of the trainee with the simulator

Nearly all the participants wanted easy access to the simulator, referring to the simulator being onsite at the hospital they work at reducing travel time to a minimum. The quantitative data in Fig. 6 corroborates the subtheme by greater variation in frequency of use (12 uses minimum to 167 uses maximum) when participants were onsite compared to when they had to travel (3 uses minimum to 69 uses maximum). A statistically significant difference between the two groups (*p* = 0.045) was found (Fig. 6). The frequency of use of the simulator was higher when there was less distance travelled to use the simulator, indicating that the qualitative and the quantitative date correlate.

#### Emphasis on engagement with simulation periodically rather than frequently may help incorporate simulation as a routine into training

**Fig. 7:**
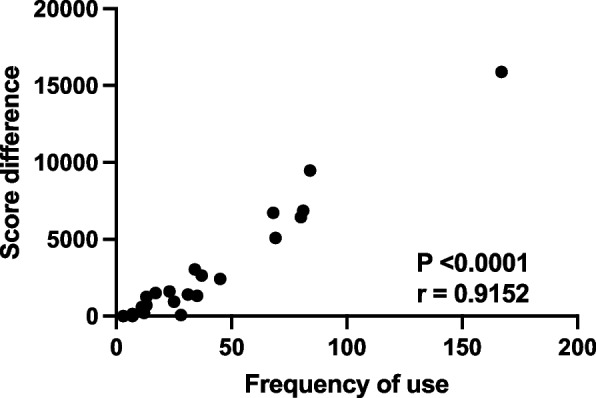
Comparison of frequency of use of the simulator to the score difference between final score and baseline score. *x* axis: frequency of use of the simulator, *y* axis: score difference. With increased frequency of use, the score increased proportionally. No participant had final scores that reduced to below their baseline score which may indicate that even with less frequent use some benefit may be seen in skills. The *p*-value from the two-tailed test is < 0.0001, suggesting a statistically significant relationship between the variables. The higher the value of frequency of use indicated better engagement of the trainee with the simulator

This subtheme was evident when analysing the interview data where participants acknowledged that whilst using simulation more would result in possibly better skills, it may not always be practical to use it very frequently, balancing use with their other time commitments. A practical approach of to using the simulator for technical skill training would be to schedule more frequent sessions when learning a skill before reducing engagement to maintain the skill. The participants did not think frequency of use of the simulator was as important as regular scheduled access to the simulator similar to being scheduled to attend the operating theatre as part of their training. The emphasis was more on periodic use with regular scheduled sessions that prompted them to engage with simulation balanced alongside their theatre and other training related sessions. The quantitative data analysis showed an increase in the overall score with all participants who used the simulator, with the exception of two participants who saw no change in score (Table 1). The paired t test comparing the mean difference in final score to the baseline score showed a statistically significant difference (*p* = 0.00064). Figure 7 compares frequency of use to score difference and demonstrates that with increased use, the score also increases. A Spearman rank correlation (Spearman’s *r*) test was conducted to assess the relationship between the frequency of use of the VR simulator and the difference in score for each participant. The analysis revealed a strong positive correlation, with a Spearman *r* value of 0.9152, suggesting that increased use of the simulator was associated with a greater improvement in scores. The 95% confidence interval for Spearman’s r ranged from 0.7990 to 0.9655, reinforcing the strength and reliability of this relationship. The *p*-value from the two-tailed test was < 0.0001, indicating that the correlation is statistically significant. Interestingly, even with less frequent use there was a positive change in the score (Table 1 and Fig. 7), which corroborates the participants’ experiences noted in the qualitative data emphasising periodic use rather than frequency (Table 2, subtheme 2).

#### Emphasising simulation in routine training is essential for trainees with limited opportunities

Limited opportunities encompassed surgical subspeciality rotations where access to a type of surgery was anticipated to be low. For example, participants felt they could maintain laparoscopic skills by practising on a simulator when they were working in a subspeciality where open surgery was the norm. On the flip side, practicing on a simulator to become proficient in laparoscopic suturing before they rotated into the upper gastrointestinal surgery department was described as a preparatory step that helped them assimilate and impress their consultant from the start.

#### Choosing the correct motivating goal- achieving specific scores for each exercise, participating in “leader board” style challenges and individualised training plans may be more effective than arbitrary repetition/engagement target

The participants’ collective experience with the various forms of simulation they had been exposed to which included a box trainer, VR simulator in this study, AR simulator and the robotic simulator helped them compare the various goals they had been set over the course of their training to decide the motivators that helped them engage with simulation in a meaningful way. A structured programme (as with the VR study), with a target score to be achieved in each exercise rather than an arbitrary repetition of exercise (like with the robotic simulator with a target score of 90% before they could move on to the next exercise) and a gamified approach appealing to the competitive nature of the surgical participant (with a leader board type display) were all highly motivating to encourage engagement.

#### ARCP oversight of simulation engagement motivates trainees to increase their usage

Participants were encouraged to engage with simulation when it was incorporated into the targets to be achieved as part of their regular training and included discussion and feedback with consultants, in the same way they would about real-life patients. They wanted to be held accountable by the ARCP process and wanted to track their simulation progress over time as they would with their other training goals like the case logbook. Personalisation of their goals should include simulation targets, where specific skills could be identified for practice on a simulator that were individualised based on the evidence of progress they have collected and reviewed at ARCP.

### Theme 2: Early introduction of simulation in surgical training (Table 3)

**Table 3:** Theme 2: Early introduction of simulation in surgical training and the 3 subthemes

Subtheme	Example excerpts from questionnaires (Tr-trainee/participant)	Example excerpts from follow-up interviews (Tr-trainee/participant)
1. Simulation training should be implemented from the start of surgical education (CT1 onwards)	Tr 1: “Early stages for skill development, later stages if new more complex modules” Tr 3: “Very useful for CT1-CT2 for manual skills, muscle memory, hand–eye coordination. ST3-ST4 would benefit as well as they still in early stages and don’t have much experience. ST5-6 would need more complex exercises” Tr 5: “CT1-ST4” Tr 6: “Core trainees” Tr 7: “Early-stage trainees for improving hand eye coordination and assisting at laparoscopy by holding the camera as well as simple operations like the appendix” Tr 12: “Core training and below to develop laparoscopy skills including camera holding” Tr 22: “I think currently this would be most useful at core training level for an introduction to basic operations. There are benefits at all levels in terms of hand–eye coordination and economy of movement, but I’m not convinced this is the best way to acquire these skills.”	Tr 1: “Now we are pushing more towards robotic and laparoscopic. I think in the future; simulation will probably play a bigger part and will be important to start early” Tr 3: “Simulation is useful for people who haven’t done anything in theatre or don’t have much experience. When they progress to core training, they are very keen to operate appendices or diagnostic laparoscopies, so for trainers its quite good to have already trained people for the basic skills.” Tr 4: “Surgical training starts officially at CT1. So that’s when u start.” Tr 6: “I come in at ST1 or CT1. I want to learn or do an appendix. I have learnt the cognitive steps of doing an appendicectomy and I’ve done this many simulations on a model–3 or 4, just to kind of really consolidate the cognitive steps in my head and I train here, and I go and do an appendix on a real human being supervised”
2. It is easier to teach trainees using simulation first before progressing to real life operating	Tr 4: “Going through the steps of an operation before doing it in real” Tr 11: “I believe this simulator would be good for novice laparoscopic surgeon such as core surgical trainee or ST3 level to familiarise themselves with steps of surgery and dealing with potential complications” Tr 13: “Good at teaching the basic steps of the operation” Tr 16: “Helps build awareness of operative steps” Tr 21: “It is good at learning the steps of the operation, understanding port placement and developing hand eye coordination”	Tr 4: “As a trainer I would be much more comfortable with trainees using a simulator. You don’t want to be training someone who takes 15 min to load a needle and then you don’t want them to put it somewhere (sic)” Tr 5: “Like when you first learn to stitch skin, I was first taught by someone to do that on a model. So, it seems bizarre that we then learn to do the steps of a gall bladder on patients when there is nothing in between” Tr 7: “The robotic surgery is useful because you are suddenly using your feet for changing things, again I think for confidence and knowing that what you are going to press is ok and that suddenly the robot is not going to do something unexpected, definitely have to do the simulator before real life”
3. Combined technical and nontechnical skills simulation would offer a more naturalistic setting to practice the skills necessary for real world operating	Tr 4: “If the focus was not on technical performance, but overall performance in a stressful scenario, e.g. bleeding lap cholecystectomy while junior comes into theatre to say someone is sick on ward etc. then that could be helpful” Tr 7: “Yes, though I have never participated in this type of simulation before” Tr 9: “Yes, I think that this would be possible” Tr 11: “Yes, This idea could be useful for management of unexpected intra-operative complications” Tr 12: “Yes this would be an excellent use but the obvious difficulties with cost and setup of these scenarios applies to this sim as to others”	No corresponding interview data as this question was not discussed during the interviews

#### Simulation training should be implemented from the start of surgical education (CT1 onwards)

This was a unanimous finding when discussing all types of technical skills simulation but especially laparoscopy related simulation. All of the participants felt simple exercises that help develop hand–eye coordination, improve camera manoeuvring skills, economy of movement and tissue handling were essential to engage with from the start of surgical training. They did not perceive a benefit starting earlier than CT1. The participants liked the modules on the VR trainer for practising the steps in the actual sequence of a basic operation like an appendicectomy before coming to theatre. They also appreciated the usefulness of this experience for those starting out in training.

#### It is easier to teach trainees using simulation first before progressing to real life operating

Some of the senior participants did not personally feel they gained a lot from using the simulator. These participants, however, recognised the value of teaching using the simulator, especially as they transitioned from trainee to consultant in the immediate future. The collective view was to normalise using simulation to learn a technical skill, even if the skill formed a part of a bigger operation (e.g. suturing) before the trainee performed it on a patient. There was value in simulation teaching the cognitive steps involved with performing the technical skill as well as for participants to learn the overall steps involved in the operation, making it easier for trainers and consultants to teach them in real life when they came to the operating theatre.

#### Advancing technical skills development through combined technical and non-technical skills simulation is desired

None of the participants had participated in a simulation that combined technical and non-technical skills. Their experience with non-technical skill simulation was restricted to attending courses like the Advanced Trauma Life Support (ATLS) occasionally during their training and they did not report participating in these training sessions during their regional training days. The participants could see the value of simulating a real-life emergency in theatre or on call where technical skills were required whilst maintaining communication and situational awareness with the wider team. However, they had never participated in such a simulation before and did not have a mental model of the experience.

## Discussion

This case study explored the experiences of general surgical trainees in the Thames Valley deanery during and after their engagement with a VR laparoscopic simulator which was implemented as an adjunct to their regular training using a structured programme. The results have provided valuable insights into the motivations, challenges, and preferences of the trainees with regard to regular engagement with simulation for technical skill training.

### Varied motivators encourage engagement with simulation

Trainees, depending on their learning styles, access to theatres, time commitments and stage of training are motivated by different reasons to engage with simulation training. This study highlighted the importance of ease of access to simulation as a common motivating factor that demonstrated increased use of the simulator. This evidence is corroborated by previous studies on the increasing the use of simulation [33]. But beyond access to a simulator, this study highlighted several factors that could be introduced, at no additional cost, to sustain engagement in the long term, irrespective of the simulator. These include short term targets trainees must achieve using simulation that are determined based on their long-term individual learning goals, having trainer and ARCP accountability at least annually to discuss their simulation engagement and the emphasis on distributed and deliberate use of simulation especially if low access to surgical time is anticipated. Previously conducted studies have focussed on the use of simulation in study conditions in isolation of routine training [33, 39]. Performance goals have been highlighted as a motivator in surgical training which contributes further to the concept of proficiency based surgical training, though it has not been extensively studied [40–42].

The emphasis on periodic, but predictable, rather than frequent simulation sessions is a new finding to the existing recommendations regarding technical skills surgical simulation. Whilst traditionally studies have recommended frequent use referring to more engagement resulting in better skills (without a specific recommendation of time spent practicing on a simulator), it is not practical given the time commitments trainees have to balance [27]. A pragmatic approach the trainees in this study suggested is for sessions to be scheduled periodically over their placement to balance them with other training commitments. Our findings is supported by existing literature that has demonstrated the superiority of distributed practice over “bootcamp” style massed practice [43, 44].

On the other hand, previous research has shown low engagement with using unrestricted access to simulators without learning goals [33]. The focus on learning specific skills during these sessions can be determined by their overall learning goals, contributing to a holistic approach to technical skills training that views simulation as an adjunct to skills a trainee learns in the operating theatre. The positive correlation between simulation use and improved scores, even with less frequent sessions, underscores the importance of consistency in engaging with training alongside clinical work.

Trainees in this study acknowledged the value of simulation in maintaining and developing skills, particularly when opportunities for hands-on practice in certain surgical subspecialties were limited. Though the trainees discussed subspecialities that did not use certain surgical techniques like laparoscopy to indicate limited opportunities, the findings can be extrapolated to training situations where there is global reduction in operating like going out of training for research or maternity leave or working less than full time, where skill maintenance is an important parameter to consider [45, 46].

Trainees expressed their desire to use simulation to prepare for specific subspeciality rotations ahead of time so that they could make a positive impression during their first theatre session. Laparoscopic suturing was one of the skills the trainees could see the value of practicing before they had to perform the task in theatre on a patient, as previous noted in various studies [47–49]. This preparatory use of simulation could be further encouraged as a standard practice, particularly in rotations with limited exposure to certain procedures.

### Role of choosing the appropriate goals: achievement of a set score for each exercise, gamification of simulation engagement

The study also sheds light on the effectiveness of structured goals and gamification in enhancing engagement with simulation. Trainees reported that achieving specific scores or participating in leader-board style challenges were more motivating than arbitrary repetition of tasks, appealing to the healthy competitive nature of the trainees. Gamified simulation has been studied in non-surgical contexts and has been found effective to motivate use [50, 51].

### Incorporating simulation in the ARCP review

The incorporation of simulation progress into formal assessments provides some accountability for both trainers and trainees [32]. Trainers would be held accountable to assist the trainee in setting realistic and relevant goals (simulation and routine surgical training related) and trainees would be held accountable to show their engagement with the training process. Also, the deanery/training body as a whole would be accountable for the provision of local simulation facilities that are fit for purpose so that trainees can access them readily. It is routine practice for trainees to rotate between hospitals over the course of training, helping them get access to a wide range of simulators depending on local availability. This suggestion aligns with broader educational trends that simulation can play a crucial role as an effective adjunct for learning and maintaining technical skills [32, 52].

### Early introduction of simulation into routine training as an adjunct to learning in an operating theatre

The unanimous support for introducing simulation early in surgical training, particularly for developing basic laparoscopic skills, reflects the recognition of simulation as a fundamental component of modern surgical education. The trainees recognised the importance of simulation as a preferred tool to teach the next generation of surgeons, making it easier for trainers and trainees as simulation provides an environment where mistakes do not lead to a negative impact on the patient’s wellbeing [53, 54]. As one of the participants quoted, trainees are already being taught simple surgical tasks like skin suturing with simulation first before progressing to suturing in theatre, but a similar approach is not being adopted for more complex tasks like laparoscopic surgery. Simulation also allows for novice trainees to struggle and take their time with learning complex skills in a psychologically safe environment.

The potential benefits of combining technical and non-technical skills simulation were acknowledged, although none of the trainees had participated in such an integrated simulation. This gap in training suggests an area for future development. The integration of technical and non-technical skills simulation could better prepare trainees for real-life where both sets of skills are routinely required simultaneously as shown in a few studies that simulated endoscopic, theatre and ward settings [55–57]. Future research should explore the implementation of such combined simulations and evaluate their impact on trainee competence and patient outcomes.

The study further supports the idea that integrating simulation into routine training requires strategic planning to ensure that trainees can access these tools without disrupting their already demanding schedules. Certainly, in our deanery, we have initiated the process of incorporating this study’s findings into routine surgical training including working with training programme directors and surgical educators to normalise the use of simulation as a regular complementary tool for technical skills and changing the ARCP process to formalise the role of simulation engagement.

## Limitations and future directions

The sample size in this study was small (although it comprised nearly the entirety of the general surgical trainees in the ST3-8 cohort in the deanery), and the study was conducted within a single training region in the UK. The trainees in this region had exposure to various simulation modalities over the course of their training though none were used consistently. The generalisability of the findings may be affected by these factors.

Future research should focus on implementing the regular use of technical skill simulation as an adjunct in routine training. Additionally, investigating the feasibility and effectiveness of integrating simulation into the ARCP process could provide a pathway for formalizing its role in surgical education. Further work is needed to combine technical and non-technical skill simulation to enhance technical skill learning in complex environment. These strategies are aligned with the Educator Workforce Strategy developed by NHS England that emphasises the embedding of evolving and innovative models of education including the use of simulation and immersive technologies [58].

## Conclusion

This study highlights the critical role that simulation can play in technical skill surgical training, particularly when implemented as an adjunct into routine training and tailored to the individual needs of trainees. The findings suggest that ease of access, structured goals, and the early introduction of simulation are key factors in maximising its educational value. By addressing the logistical and motivational challenges identified in this study, surgical educators can enhance the effectiveness of simulation use and better prepare trainees for the complexities of real-world surgery.

## Supplementary Information

Supplementary Material 1. Baseline questionnaire.

## Data Availability

No datasets were generated or analysed during the current study.

## Abbreviations

ARCP: Annual Review of Competency Progression
ATLS: Advanced Trauma Life Support
AR: Augmented Reality
CTRG: Clinical Trials and Research Governance
CT1: Core surgical Training year 1
CT2: Core surgical Training year 2
IST: Improving Surgical Training
PGD: Postgraduate Deanery
ST3: Specialist Training year 3
ST7: Specialist Training year 7
ST8: Specialist Training year 8
UK: United Kingdom
VR: Virtual Reality
