# Supplementary material for: Exploring trainee experiences in a structured virtual reality laparoscopic training programme for general surgeons: a longitudinal case study
Source: Adv Simul (Lond). 2025 Oct 28;10:54. doi: 10.1186/s41077-025-00359-x (PMC12570560; doi:10.1186/s41077-025-00359-x)
Supplement: Supplementary file 1 — Supplementary Material 1. Baseline questionnaire. [file 41077_2025_359_MOESM1_ESM.docx]

Supplementary material

Baseline questionnaire

1. Were you on track in your training before the pandemic, with regards to the various aspects of the training?
2. Did the pandemic affect your training? How? Which areas of training are still on track and where do you think you need support?
3. What are your personal learning objectives from this simulation programme?
4. Describe the role simulation has played in your training so far. Think of the various forms of simulation- simulation to improve technical skills (e.g., laparoscopic box simulators, robotic surgery simulator), simulation to improve non-technical skills (e.g., team-based simulation) or a combination of the two.
5. How frequently have you engaged with simulation before? Give examples where possible.
6. What are the barriers in your experience to inculcating simulation into your routine?
7. Has your past experiences of simulation made an impact on your training? Expand with examples.
8. In your opinion, what are the expectations with regards to this 3-month pilot using a virtual reality simulator in combination with the home laparoscopic box trainer? Do you expect this experience will be of benefit? What do you predict will be the challenges during this programme?
9. Rate your confidence in performing a laparoscopic appendicectomy on a scale of 0-10, with 0 being the lowest confidence rating
10. Rate your confidence in performing a laparoscopic cholecystectomy on a scale of 0-10, with 0 being the lowest confidence rating
11. Rate your confidence in managing intra-operative complications (e.g., vascular injury during a laparoscopic cholecystectomy) on a scale of 0-10, with 0 being the lowest confidence rating
12. Overall, as a surgical registrar, how confident do you feel managing the on call? Rate your confidence on a scale of 0-10, with 0 being the lowest confidence rating
13. Overall, as a surgical registrar, how confident do you feel managing an elective theatre list independently? Rate your confidence on a scale of 0-10, with 0 being the lowest confidence rating
14. Do you think the pandemic has had an impact on your confidence to do your job? In which area (managing an on call, running a theatre list, performing specific surgeries, managing complications) has your confidence been affected the most? Can you quantify this change using the confidence rating scale of 0-10, with 0 being the lowest confidence rating? E.g.: I was 8/10 confident in performing this procedure before the pandemic but now I’m 6/10.
15. Using the confidence scale described above, how confident do you think a surgical trainee should be at CCT/ as a new consultant? Do you think you are on track to achieve that?
16. Do you think this simulation training will help improve your confidence rating?

Follow-up questionnaire

## Section A: Impact on individual skills with simulator use

From a scale of 1 to 10, indicate how useful the simulator was to improve this skill with 10 being the most useful. Also, please comment on the impact use of the simulator has had on each of the following skills.

Q1: Hand-eye coordination: 1 2 3 4 5 6 7 8 9 10

Q2: Economy of movement: 1 2 3 4 5 6 7 8 9 10

Q3: Learning/practicing the steps of the operation: 1 2 3 4 5 6 7 8 9 10

Q4: Planning ahead and anticipating complications: 1 2 3 4 5 6 7 8 9 10

Q5: Managing complications: 1 2 3 4 5 6 7 8 9 10

Q6: Are there any other skills this simulator training helped improve? List them below with your comments and use the scale to help us understand how useful the simulator was

1 2 3 4 5 6 7 8 9 10

## Section B: Feedback after use of the VirtaMed simulator

Q1: Frequency of simulation use- Were you satisfied with how frequently you used the simulator? Would more use have benefitted you? Expand on your answer

Q2: What was the biggest challenge for using the simulator?

Q3: What is the simulator good at, in terms of the hardware/software/ interface/programming/ exercises available?

Q4: What can be improved with the simulator? Where there any difficulties with using the machine itself?

## Section C: Potential uses for the simulator

Q1: Stage of training- In your opinion, at what stage of training (CT1,2, ST3… etc) is this simulator most useful for? Why? Or do you think there are benefits to using this simulator at any stage of training?

Q2: Could this simulator be used to assess trainee technical skills? Please expand on your answer.

Q3: Could this simulator be used as part of a combined technical skills & non-technical skill team simulation? E.g.: A scenario where you are the primary surgeon operating on the simulator where a complication arises, and you are expected to manage the complication in a simulated theatre with the wider MDT team (anaesthetists, theatre staff etc).

Q4: Were you able to practice on a department/home lap trainer box? Expand on your answer as to what the impact of this practice was? Or if you did not, what were the barriers?

## Section D: Were personal objectives met? Impact on confidence

Q1: You had listed your personal objectives for the 3-month simulation pilot. Were these expectations met? Expand on how the simulation did or did not help you meet your personal objectives.

Q2: Did the simulation training have any impact on confidence in operating/running the on call/managing complications/overall confidence?

Interview guide

Introduction:

1. Introduce myself and the purpose of the interview.
2. Explain the semi-structured nature of the interview (allowing for both pre-determined questions and the opportunity to share experiences and insights)
3. Confidentiality and informed consent
4. More detailed responses are welcome.

Section 1:

Comparing VR and AR Simulators

Goal: Compare the experience of using VR vs AR. Does the platform matter for learning?

1. Tell me about your experience of using the VR simulator.
2. How would you describe your experience using the augmented reality (AR) simulator in comparison to the VR simulator?
3. What are the main advantages of using AR for surgical training? In your opinion, what are the main limitations of the AR simulator?
4. In your opinion, in what ways does the platform (VR vs AR) make a difference in terms of learning and skill development? Please elaborate.
5. Does frequency of use of a simulator have an impact on learning? Do you think you see a benefit even when you use it infrequently?

Section 2: Structured Approach to Technical Skill Simulation

Goal: Determine the impact of a structured approach on adherence

1. Did you find the structured approach beneficial for your learning and skill development? Why or why not?
2. Did the structured approach affect your adherence to the training program? If so, in what ways?

Section 3: Impact of Simulation Training on Real-Life Practice

Goal: Assess the impact of sim training on real-life practice

1. Has simulation training had an impact on your real-life surgical practice? Can you provide examples of specific skills or techniques that you have improved or implemented as a result of simulation training?

Section 4: Ideal Integration of Technical Skill Simulation into Training

Goal: Determine trainees' perspectives on integrating sim training

1. In your opinion, what would be the ideal way to integrate technical skill simulation into the general surgical training programme? What aspects of sim training do you think could be improved or enhanced to make it more effective?
2. Are there any specific resources or support systems that you believe would facilitate the integration of sim training into the training programme?

Closing:

Do you have something else you would like to add to what we have discussed?
